# ENERGY EXPENDITURE AND PHYSICAL ACTIVITY AMONG OLDER PARTICIPANTS FROM THE MULTICENTER AIDS COHORT STUDY (MACS)

**DOI:** 10.1093/geroni/igz038.2023

**Published:** 2019-11-08

**Authors:** Jennifer A Schrack, Todd T Brown, Joseph B Margolick

**Affiliations:** 1 Johns Hopkins University, Baltimore, Maryland, United States; 2 Johns Hopkins Medical Institutions, Baltimore, Maryland, United States; 3 Johns Hopkins Bloomberg School of Public Health, Baltimore, Maryland, United States

## Abstract

Energy utilization becomes more inefficient with age and is linked to low physical activity and functional decline. Persons aging with HIV exhibit accelerated functional decline, but the effect of chronic HIV infection on energy utilization and free-living physical activity remains unclear. We investigated cross-sectional associations between age and: resting metabolic rate, peak walking energy (VO2), and 7-day physical activity by accelerometry in 100 men in the MACS (age: 60.8+/-6.8 years, 35% black, 46.1% HIV+, 94% virally suppressed). In multivariable regression models adjusted for age, BMI, race, chronic conditions, and HIV viral load, HIV+ men had a higher resting metabolic rate (β=103.2 kcals/day, p=0.03) and lower peak walking VO2 (β=-1.8 ml/kg/min, p<0.02) than HIV- men. Moreover, HIV+ men demonstrated lower physical activity, overall and by time of day (p<0.05). These results suggest that energy utilization differs by HIV serostatus, which may contribute to lower physical activity and function with aging.

